# Prognostic Role of Hypothyroidism in Heart Failure: A Meta-Analysis

**DOI:** 10.1097/MD.0000000000001159

**Published:** 2015-07-31

**Authors:** Ning Ning, Dengfeng Gao, Vincenzo Triggiani, Massimo Iacoviello, Judith E. Mitchell, Rui Ma, Yan Zhang, Huijuan Kou

**Affiliations:** From the Department of Nuclear Medicine (NN), The Second Affiliated Hospital, Xi’an Jiaotong University School of Medicine, Xi’an, Shaanxi, P.R. China; Department of Cardiology (DG, RM, YZ, HK), The Second Affiliated Hospital, Xi’an Jiaotong University School of Medicine, Xi’an, Shaanxi, P.R. China; Endocrinology and Metabolic Diseases (VT), Interdisciplinary Department of Medicine, University of Bari, Bari, Italy; Cardiology Unit (MI), Department of Emergency and Organs Transplantation, University of Bari, Bari, Italy; and State University of New York Downstate Medical Center (JE), Brooklyn, NY.

## Abstract

Hypothyroidism is a risk factor of heart failure (HF) in the general population. However, the relationship between hypothyroidism and clinical outcomes in patients with established HF is still inconclusive.

We conducted a systematic review and meta-analysis to clarify the association of hypothyroidism and all-cause mortality as well as cardiac death and/or hospitalization in patients with HF. We searched MEDLINE via PubMed, EMBASE, and Scopus databases for studies of hypothyroidism and clinical outcomes in patients with HF published up to the end of January 2015. Random-effects models were used to estimate summary relative risk (RR) statistics. We included 13 articles that reported RR estimates and 95% confidence intervals (95% CIs) for hypothyroidism with outcomes in patients with HF. For the association of hypothyroidism with all-cause mortality and with cardiac death and/or hospitalization, the pooled RR was 1.44 (95% CI: 1.29–1.61) and 1.37 (95% CI: 1.22–1.55), respectively. However, the association disappeared on adjustment for B-type natriuretic protein level (RR 1.17, 95% CI: 0.90–1.52) and in studies of patients with mean age <65 years (RR 1.23, 95% CI: 0.88–1.76).

We found hypothyroidism associated with increased all-cause mortality as well as cardiac death and/or hospitalization in patients with HF. Further diagnostic and therapeutic procedures for hypothyroidism may be needed for patients with HF.

## INTRODUCTION

Heart failure (HF) is a principal complication of all forms of heart disease.^1^ Approximately 10 million people in the United States and Europe have chronic HF, and 1 million patients receive a diagnosis of HF each year.^2^ Being one of the principal causes of morbidity, mortality, and hospitalization, HF represents a major public health care and economic problem around the world. In a population-based cohort, survival after diagnosis of HF was 63% at 1 year and 35% at 5 years.^3,4^ Recognizing modifiable risk factors for HF outcomes is essential to target subjects at risk of developing this condition.^5^

Neuroendocrine activation is important in the progression of HF. Increasing studies have indicated that thyroid hormone may play an essential role in maintenance of cardiovascular homeostasis under physiologic and pathologic conditions, and is involved in modulating cardiac contractility, heart rate, diastolic function, and systemic vascular resistance,^6,7^ thereby affecting cardiac function. Moreover, thyroid dysfunction is a risk factor of cardiovascular disease as well.^8–10^

Hypothyroidism, defined as an elevated serum level of thyroid stimulating hormone (TSH), with free thyroxine (FT4) or triiodothyronine (FT3) levels within the reference range (subclinical hypothyroidism) or below the reference range (overt hypothyroidism),^11^ is common in the general population.^12^ Studies comparing outcomes with hypothyroidism and euthyroidism (normal thyroid gland function) in the general population found hypothyroidism independently associated with mortality.^13–15^ Moreover, a pooled analysis of individual patient data for 6 prospective cohorts with thyroid function tests and follow-up of HF events demonstrated an increased risk of HF events in patients with hypothyroidism.^8^ However, only a few studies have investigated the effect of hypothyroidism on all-cause mortality in patients with cardiac disease and specifically HF. Some studies described an increased risk of all-cause mortality for HF patients with hypothyroid TSH level,^16,17^ but others did not.^18^ In most of the studies, the number of patients with hypothyroidism was small, and the findings may lack statistical power. Thus, the prognostic impact of hypothyroidism in HF is still inconclusive and partly conflicting.

The purpose of this meta-analysis was to clarify the association between hypothyroidism and outcomes including death and hospitalization in patients with HF using data from all available prospective studies.

## DATA SOURCES AND SEARCH STRATEGY

We performed a systematic literature search of the association of hypothyroidism and mortality (cardiovascular and total) and/or cardiac hospitalization. We searched MEDLINE via PubMed (publications from 1966 to May 2014), EMBASE (publications from 1980 to May 2014), and Scopus (www.scopus.com), with no restriction of language. We used combinations of text words and thesaurus terms that included TSH, thyrotropin, hypothyroidism, heart failure [MeSH], and the following keywords: subclinical hypothyroidism, hypothyroidism, subclinical dysthyroidism, and subclinical thyroid. We also searched the reference lists of all studies included and those from published systematic reviews.

## STUDY SELECTION

Studies were included if they were clinical or cohort studies, involved adults (≥18 years old), and investigated the relation between hypothyroidism and outcomes in patients with HF. All abstracts were scanned independently by 2 investigators (DG and NN), who then retrieved the full text of potential articles. Disagreements were resolved by consensus, and if necessary, a third author (HK) was required to assess it. To be included in the analyses, studies had to provide estimates of relative risk (RR) (such as odds ratios [ORs], hazard ratios [HRs], or risk ratios) with 95% confidence intervals (95% CIs). If the article lacked data, we attempted to contact the author.

## DATA EXTRACTION AND QUALITY ASSESSMENT

We designed a data collection form before the search. Two independent reviewers used this form to extract the relevant information from the selected studies (RM and HK). Any disagreements were resolved by discussion. Two independent reviewers (DG and NN) evaluated the quality of the studies included by using a modified scoring system that was created on the basis of a recently developed system (designed with reference to QUATSO, MOOSE, and STROBE) that allowed a total score of 0 to 7 points (7 reflecting the highest quality)^19^ for the following criteria: methods of outcome adjudication and ascertainment accounted for confounders and completeness of follow-up ascertainment; study populations considered a convenience or a population-based sample; appropriate inclusion and exclusion criteria; thyroid function measured more than once; methods of outcome adjudication categorized as use of formal adjudication procedures and adjudication without knowledge of thyroid status; adjustments made for age, sex, New York Heart Association (NYHA) classification, left ventricular ejection fraction (LVEF), and medication; and any other adjustments (such as for B-type natriuretic protein [BNP] level, amiodarone use, and concomitant medication for HF).

## STATISTICAL ANALYSIS

HRs and RRs were assumed to be approximately the same measure of RR. For articles reporting ORs, we estimated the RRs from the ORs using a previously published correction method.^20^ To calculate summary estimates and 95% CIs of the risk of hypothyroidism, we pooled RRs by random-effects models. The natural logarithm of the RR from each study was weighted by the inverse of its variance and pooled across studies. A 2-tailed *P* < 0.05 was considered statistically significant. Heterogeneity among studies was assessed by I^2^. I^2^ > 50% indicated at least moderate statistical heterogeneity.^21^ The amount of total variation explained by the between-study variation, and the *Q* test with *P* = 0.10. To explore sources of heterogeneity, we conducted subgroup and random effects univariate and multivariate meta-regression with a random-effects model.^22^ We also performed a sensitivity analysis by excluding 1 study at a time to assess the effect of individual studies on the summary estimates. Stata 12.0 (Stata Corp, College Station, TX) was used for statistical analyses. *P* < 0.05 was considered statistically significant.

## RESULTS

### Study Selection

**FIGURE 1:**
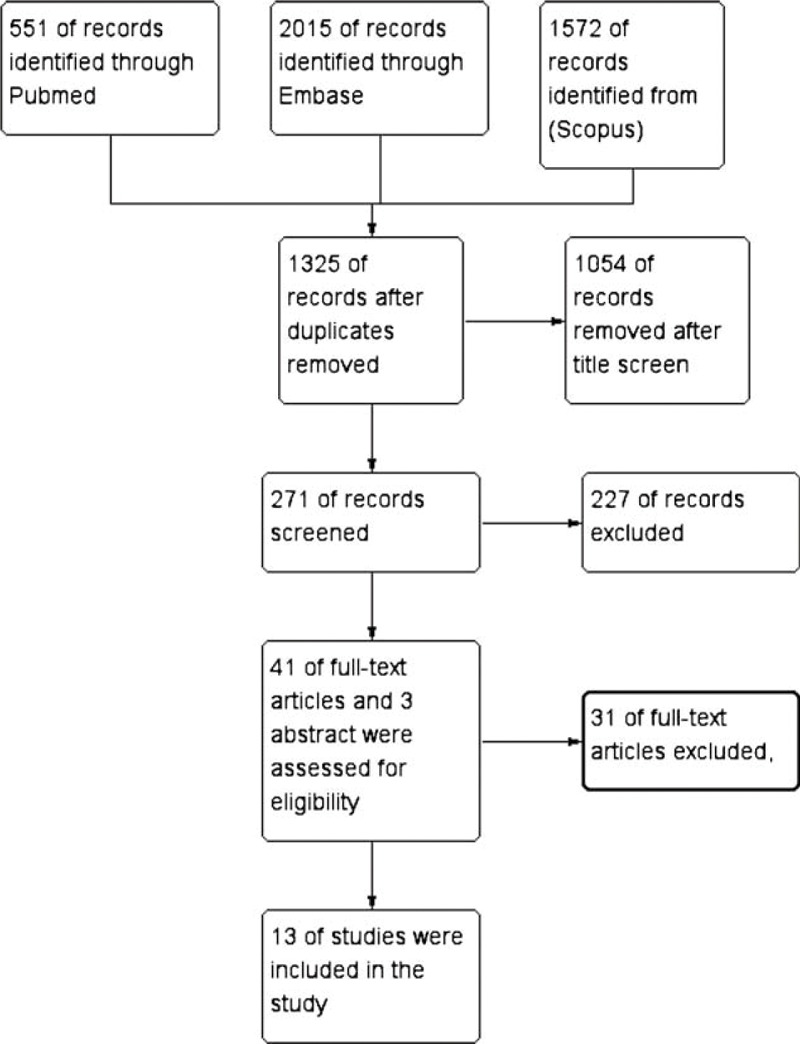
Flow chart of the selection of studies.

Of the 1325 reports identified, we excluded 1282 that were unrelated to the association of hypothyroidism and clinical outcome in patients with HF and another 30 after detailed evaluation (Figure 1). We included 13 prospective studies^16–18,23–32^ in our analysis. The articles were published between 2008 and 2015, and included 20,446 subjects (2307 cases with hypothyroidism). The mean prevalence of hypothyroidism was 11.4% (range 4%–24%).

### Description and Quality of the Studies

Characteristics of included studies are shown in Table 1  . The time to follow up ranged from 4 months to 3.5 years. The mean study population was 1572 (range 122–5599). The definition and method of assessing hypothyroidism varied across studies: TSH levels in 6 studies^16–18,25–27^ and the full spectrum of thyroid hormone levels in 7 studies.^23,24,26,27,29–31^ In total, 10 studies^16–18,24–30^ reported all-cause mortality, and 10^16–18,23,24,26,28,30–32^ reported cardiac mortality and/or hospitalization. Most of the studies used at least age and sex for adjustment. However, the number of variables differed significantly among the studies.

**TABLE 1:**
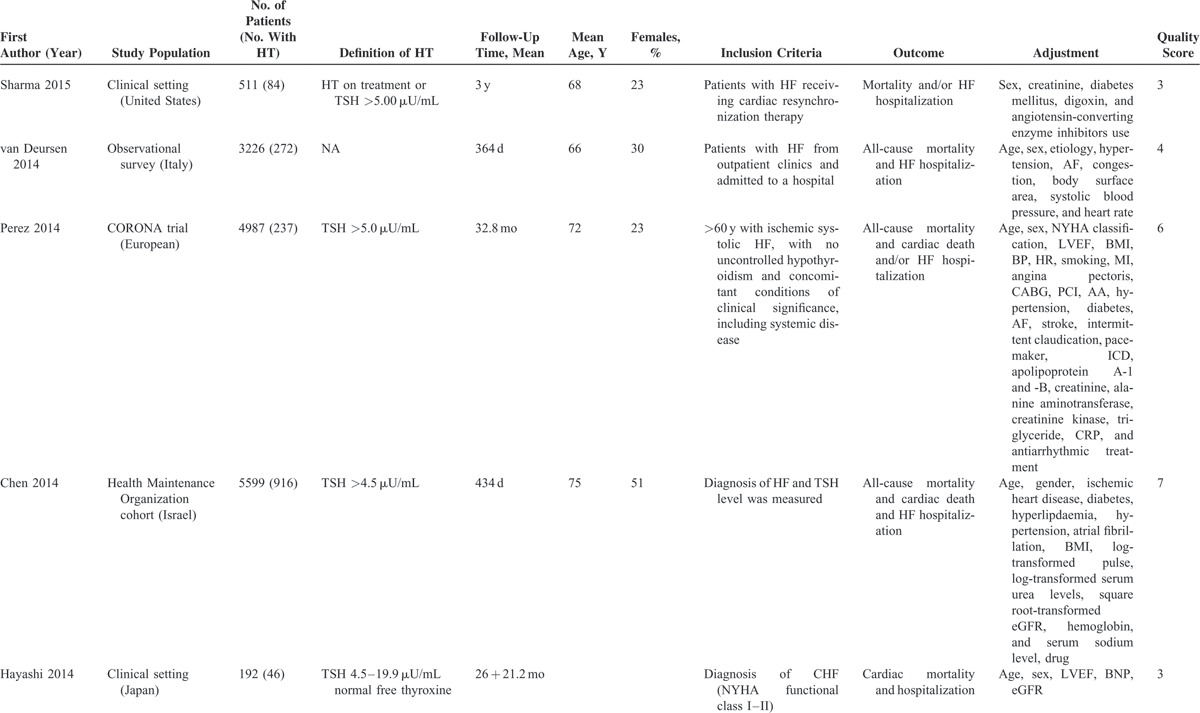
Characteristics of Studies of Hypothyroidism (HT) and Outcomes in patients with Heart Failure (HF) Included in the Meta-Analysis

**TABLE 1 (Continued):**
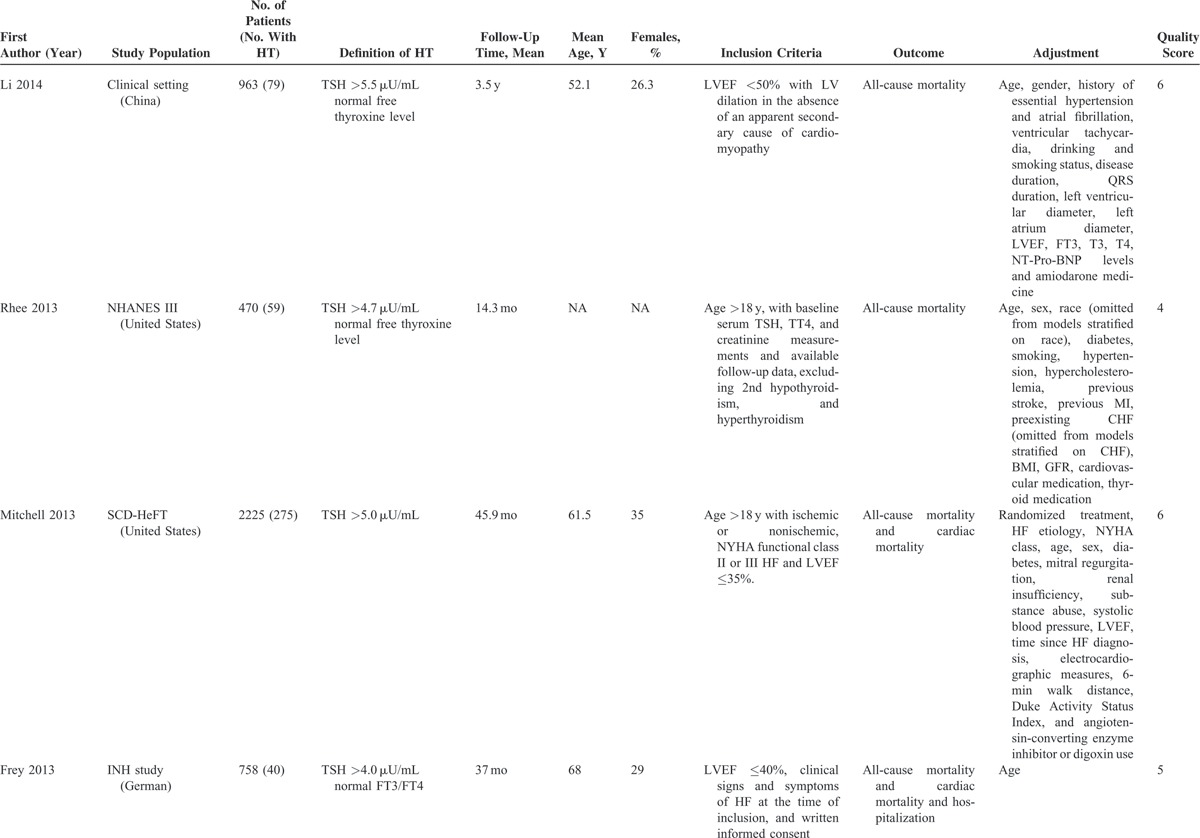
Characteristics of Studies of Hypothyroidism (HT) and Outcomes in patients with Heart Failure (HF) Included in the Meta-Analysis

**TABLE 1 (Continued):**
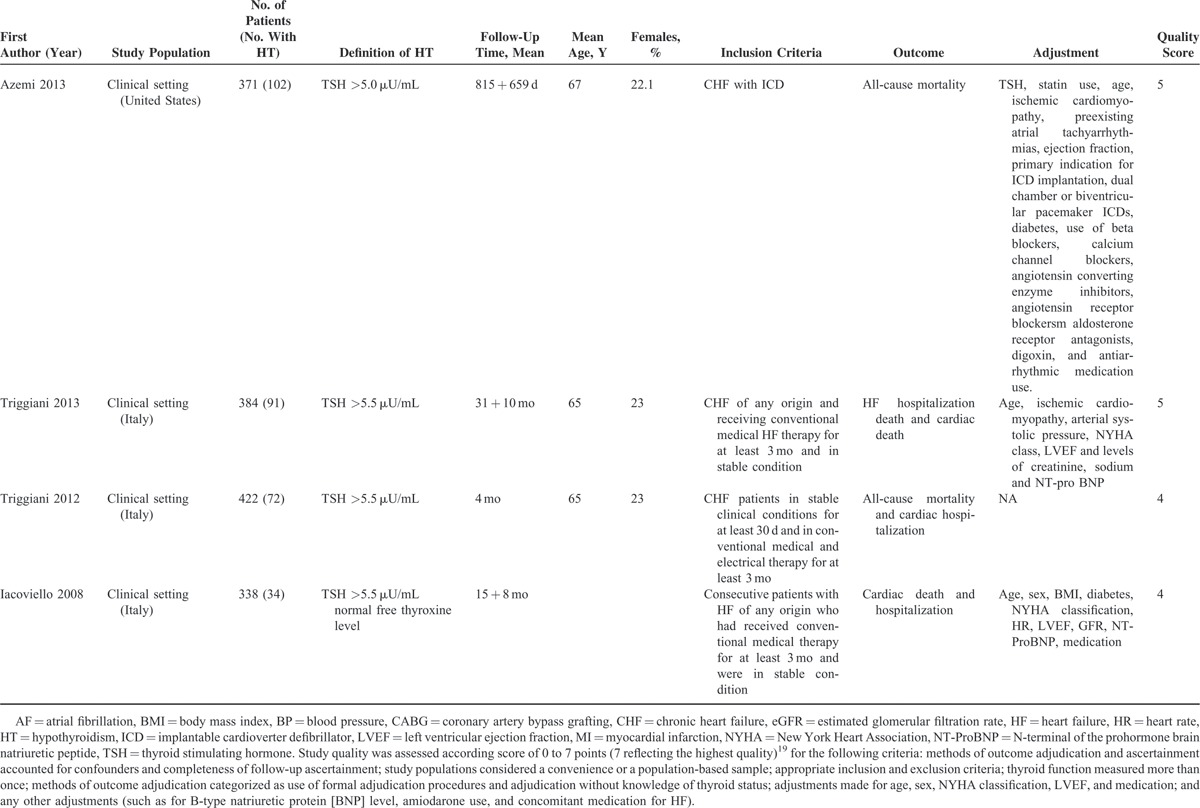
Characteristics of Studies of Hypothyroidism (HT) and Outcomes in patients with Heart Failure (HF) Included in the Meta-Analysis

### Hypothyroidism and All-Cause Mortality in Patients With Heart Failure

**FIGURE 2:**
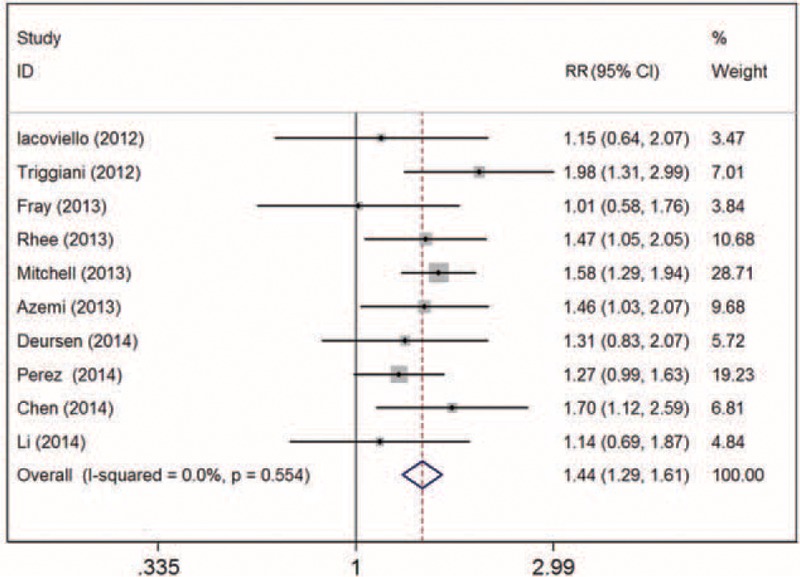
Forest plot of relative risk (RR) for hypothyroidism and all-cause mortality in patients with heart failure. Weights are from random-effects analysis.

**FIGURE 3:**
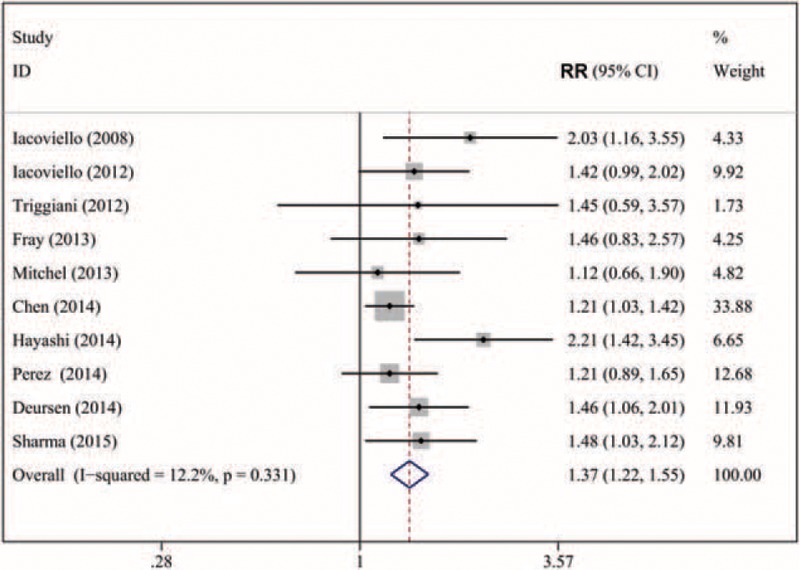
Forest plot of RR for hypothyroidism and cardiac death and/or hospitalization in patients with heart failure. Weights are from random-effects analysis.

In all, 10 studies^16–18,24–30^ including 19,354 subjects with HF (2173 with hypothyroidism) were analyzed. As compared with patients with euthyroidism, for patients with hypothyroidism, the overall multivariable-adjusted RR for all-cause mortality was 1.44 (95% CI: 1.29–1.61; *P* for heterogeneity = 0.554, I^2^ = 0) (Figure 2). On sensitivity analysis, the combined RRs all showed statistical significance, with a narrow range from 1.39 (95% CI: 1.22–1.1.58) to 1.49 (95% CI: 1.31–1.68). We also conducted a subgroup analysis to evaluate the effect of methodological and study characteristics on RR of all-cause mortality (Table 2). For studies defining hypothyroidism solely on TSH level,^16–18,25–27^ hypothyroidism was still associated with all-cause mortality (RR 1.47, 95% CI: 1.29–1.66; *P* for heterogeneity = 0.49, I^2^ = 0). Hypothyroidism overall was positively associated with all-cause mortality (RR 1.47, 95% CI: 1.31–1.65; *P* for heterogeneity = 0.51, I^2^ = 0). In studies reporting the full spectrum of thyroid hormone levels,^23,24,26,27,29–31^ subclinical hypothyroidism^24,26,27,29^ was associated with all-cause mortality for patients with HF (RR 1.41, 95% CI: 1.15–1.74, *P* = 0.44). After excluding patients with baseline thyroid replacement therapy^16–18,24,26,27,29^ or amiodarone intake,^16,18,24,25,27,29^ the association remained (RR 1.38, 95% CI: 1.19–1.59; *P* for heterogeneity = 0.72; RR 1.37, 95% CI: 1.21–1.63; *P* for heterogeneity = 0.43, respectively). However, for studies adjusting for BNP level,^18,29,30^ the positive association disappeared (RR 1.17, 95% CI: 0.90–1.52; *P* for heterogeneity = 0.24). According to the meta-regression analyses, the association was not predicted by cutoff level of TSH (*P* = 0.21), mean TSH level (*P* = 0.54), mean age (*P* = 0.80), follow-up time (*P* = 0.49), baseline LVEF (*P* = 0.15), number of cases (*P* = 0.72), or sex (*P* = 0.34). We found no evidence of publication bias by Egger test (*P* = 0.27), Begg test (*P* = 0.76), or funnel plot (Figure 3).

**TABLE 2:**
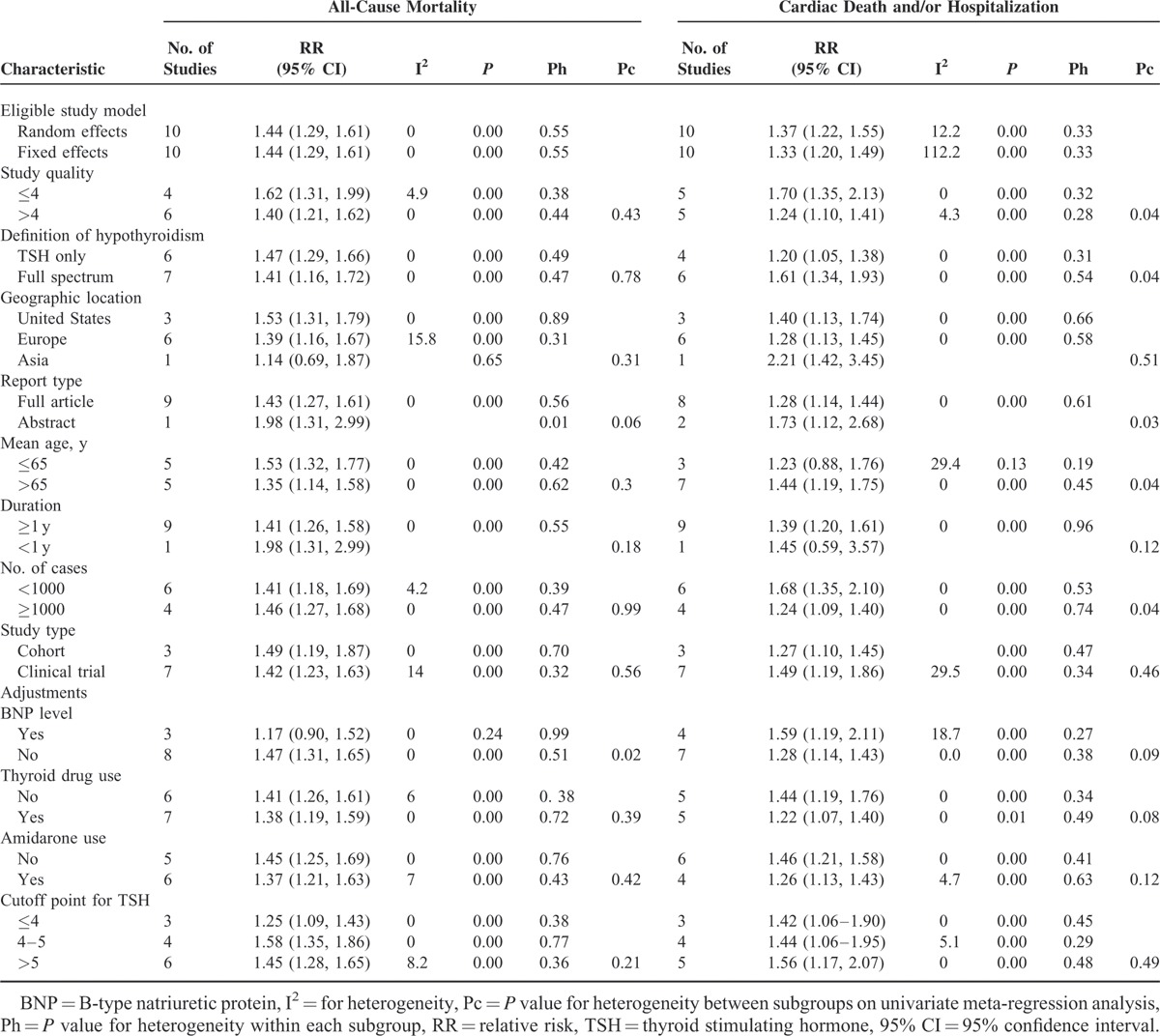
Subgroup Analysis of Hypothyroidism and Clinical Outcomes—All-Cause Mortality and Cardiac Death and/or Hospitalization—in Patients With Heart Failure

### Hypothyroidism and Cardiac Mortality and/or Hospitalization in Patients With Heart Failure

**FIGURE 4:**
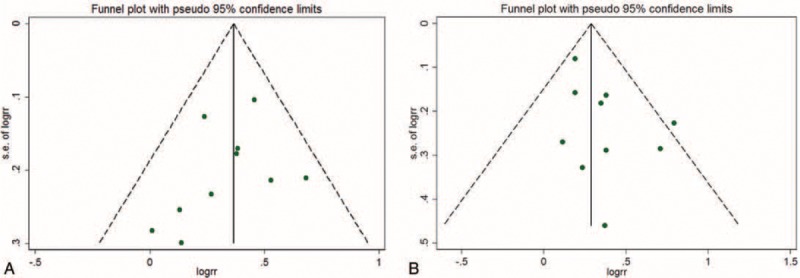
Funnel plot assessing publication bias: (A) all-cause mortality; (B) cardiac death and/or hospitalization.

We analyzed 10 studies^16–18,23,24,26,28,30–32^ including 21,858 subjects with HF (2199 cases with hypothyroidism) for and association of hypothyroidism and cardiac mortality and/or hospitalization. The overall multivariable-adjusted RR was 1.37 (95% CI: 1.22–1.55; *P* for heterogeneity = 0.33, I^2^ = 12.2%) (Figure 4). The pooled RR estimate with the fixed-effects model was 1.33 (95% CI: 1.20–1.49). In total, 4 studies^17,18,26,30^ reported the relationship between hypothyroidism and cardiac death. As compared with patients with euthyroidism, for patients with hypothyroidism, the RR was 1.36 (95% CI: 1.12–1.66; *P* for heterogeneity = 0.65, I^2^ = 0%). A total of 4 studies^18,24,30,32^ reported the relationship between hypothyroidism and hospitalization for HR. As compared with patients with euthyroidism, for patients with hypothyroidism, the RR was 1.48 (1.21–1.71; *P* for heterogeneity = 0.32, I^2^ = 11%). On sensitivity analysis, the combined RRs all showed statistical significance, with a narrow range of RR from 1.29 (95% CI: 1.16–1.44) to 1.46 (95% CI: 1.26–1.70). We also conducted a subgroup analysis to evaluate the effect of methodological and study characteristics on the RR of cardiac mortality and/or hospitalization (Table 2). For studies reporting a full spectrum of thyroid hormone levels,^15,23,26,30,31^ hypothyroidism was associated with cardiac mortality and/or hospitalization in patients with HF (RR 1.61, 95% CI: 1.34–1.93, *P* = 0.60). For studies defining hypothyroidism solely on TSH level,^16–18^ hypothyroidism was positively associated with cardiac mortality and/or hospitalization: RR 1.20 (95% CI: 1.05–1.38, *P* < 0.001; *P* for heterogeneity = 0.96). Three studies^16,17,24^ reported an association between new-onset hypothyroidism and outcomes in patients with HF. The overall multivariable-adjusted RR for composite outcomes (all-cause or cardiac mortality and/or hospitalization for HF) was 1.53 (95% CI: 1.19–1.96, *P* = 0.001; *P* for heterogeneity = 0.16, I^2^ = 41.8%). According to the meta-regression analyses, study quality (*P* = 0.04), definition of hypothyroidism (*P* = 0.04), and number of cases (*P* = 0.04) were predictors of the interaction, but not cutoff TSH level (*P* = 0.80), mean TSH level (*P* = 0.43), mean age (*P* = 0.12), follow-up time (*P* = 0.34), baseline LVEF (*P* = 0.21), number of cases (*P* = 0.41), or sex (*P* = 0.54). We found no evidence of publication bias by Egger test (*P* = 0.46), Begg test (*P* = 0.45), or funnel plot (Figure 3).

## DISCUSSION

This meta-analysis of 13 studies demonstrated that hypothyroidism, as well as subclinical hypothyroidism, was associated with substantially increased risk of all-cause mortality as well as cardiac death and/or hospitalization in patients with HF. In addition, this positive association was retained when excluding patients who used thyroid medications (mainly thyroxine replacement) or amiodarone at baseline. Further adjustment for other available HF confounding risk factors, except BNP level, did not significantly change the association with all-cause mortality and cardiac mortality and/or hospitalization.

Hypothyroidism, characterized by elevated serum TSH levels with FT4 or FT3 within the reference range (subclinical hypothyroidism) or below the range (overt hypothyroidism), is common in the general population. Population surveys reported a prevalence of 4% to 10%.^16,33^ In our meta-analysis, we found a mean prevalence of 11.4% (range 4%–24%). The large range might be explained by the difference in definition of hypothyroidism and the large cutoff value for TSH. The higher prevalence may indicate that hypothyroidism is frequent in patients with HF and may be attributed in part to amiodarone treatment.^24^ The high prevalence may be due to low-T3 syndrome (<3.1 pmol/L) in patients with HF, in that some studies define hypothyroidism by TSH level only.^34^

Studies tracking all-cause mortality as a function of thyroid status in the general population have reported markedly disparate results. Three meta-analyses assessed the risk of all-cause mortality in patients with hypothyroidism. Razvi et al^35^ and Ochs et al^13^ found total mortality more prevalent in patients with subclinical hypothyroidism. However, a recent meta-analysis, by Rodondi et al^36^, indicated no significantly increased risk of total mortality in patients with subclinical hypothyroidism. The conflicting results for all-cause mortality in patients with hypothyroidism may reflect the heterogeneity of the different studied populations in terms of sex, age, degree of TSH suppression, and duration of follow-up. Only a few studies investigated the effect of hypothyroidism on all-cause mortality in patients with cardiac disease and, specifically, HF. Some studies described elevated risk of all-cause mortality for HF patients with hypothyroid TSH levels, but others did not. In most studies, the number of patients with hypothyroidism was small, and the findings in such studies may lack statistical power. By pooling all available evidence, we found that subclinical hypothyroidism as well as hypothyroidism overall were independent predictors of all-cause mortality in patients with HF. The positive association of hypothyroidism and all-cause mortality remained on subanalysis by stratification, although in some analyses the association was not statistically significant (Table 2).

Our stratified analysis of BNP level revealed a positive correlation of hypothyroidism and all-cause mortality only in studies that did not adjust for BNP level. This finding has some explanations. First, HF alone can lead to downregulation of thyroid hormone signaling.^6^ Second, hypothyroidism may directly alter the plasma concentration of BNP.^37,38^ However, elevated TSH level may cause and aggravate HF by inducing BNP secretion and 3-hydroxy-3-methyl-glutaryl–CoA reductase (HMG-CoA) upregulation via the cyclic adenosine monophosphate (cAMP)/protein kinase A/phosphorylated cAMP response element-binding protein signaling pathway,^39^ which indicates a direct association of BNP and TSH levels. Finally, only 3 of our studies, of 407 hypothyroidism patients, adjusted for BNP level, so the findings may due to chance.

Hypothyroidism is a risk factor of HF in the general populations in various studies. A meta-analysis of 25,390 patients from 6 prospective studies showed that hypothyroidism was associated with increased risk of HF events.^8^ However, the relationship between hypothyroidism and clinical outcomes in patients with established HF is uncertain. In 2008, Iacoviello et al^23^ reported greater chance of HF progression in patients with subclinical hypothyroidism than euthyroidism. Since then, inconsistent results have been reported. Here, we found that hypothyroidism overall as well as subclinical hypothyroidism were independent predictors of hospitalization for HF and/or cardiac death. Moreover, we explored various subgroups of study design and patient demographics to investigate the reason for the differences in results among the various studies. We found that hypothyroidism was associated with cardiac death and/or HF hospitalization only in studies of patients with mean age >65 years. Thus, age might affect the association of hypothyroidism and HF outcomes. In older patients, hypothyroidism may have a severe pathophysiological effect, for accelerated HF. Alternatively, hypothyroidism may contribute equally to vascular risk at all ages, but elderly patients may feature a relatively larger component of conventional, nonhypothyroidism, vascular risk factors, and the effects of hypothyroidism may be relatively enhanced by this larger contribution of other risk factors. The existing studies may simply not have enough power to detect a relatively small contribution to vascular risk in this age group. Finally, our findings might represent a finding due to stochastic factors, because few studies of younger patients were included.

Increasing evidence suggests that thyroid hormones affect the circulation system, and the occurrence of thyroid dysfunction can lead to significant hemodynamic alterations that may cause HF events as well as worsen the prognosis of HF in patients who are already affected.^6^ The role of low thyroid hormone function in promoting HF and the potential benefits of thyroid hormone replacement have been reviewed extensively.^6^ When thyroid hormone production wanes, the organs, including the heart, demand less oxygen. The heart does not contract as strongly. It pumps less blood with every beat and refills itself incompletely. With less thyroid hormone to relax the cells that line the arteries, blood pressure in the arms and legs increases when the heart is resting between beats.^40^ In this regard, HF can lead to downregulation of the thyroid hormone signaling system in the heart. In the failing heart, decreased nuclear thyroid hormone receptor levels occur. In addition, serum levels of T4 and T3 are decreased with HF in the context of the nonthyroidal illness syndrome.^41^

Most adults with an abnormal TSH level have subclinical and not overt thyroid dysfunction.^8^ The American College of Cardiology and American Heart Association guidelines for HF support performing thyroid function tests in patients with HF, especially measuring TSH level, in recognition of hypothyroidism as a primary or contributory cause of HF.^42^ Here, we found that hypothyroid TSH level and subclinical hypothyroidism were associated with increased all-cause mortality and cardiac mortality and/or hospitalization in patients with HF. TSH levels likely also affect the outcome in patients with established HF because TSH may have extrathyroidal effects. TSH can induce interleukin 6 and tumour necrosis factor secretion in vitro^16,43,44^ and may be involved in modulating vascular function by increasing nitric oxide metabolites in vivo,^45^ which suggests a possible link between increased TSH level and inflammation as well as oxidative stress.

Our analysis had several limitations. First, we may have missed relevant research papers, thus resulting in selection bias. Second, only TSH levels were reported in some of the studies, so we could not examine the prevalence of low-T3 syndrome and its effects. We may have underestimated the potential effect of thyroid abnormality by not considering this “hypothyroid-like equivalent.” Third, most of the studies included were clinical trials, which may have had specific inclusion and exclusion criteria. Furthermore, clinical trials always recruit relatively healthy patients with less comorbidity and exclude relatively older adults, which may have led to underestimation of the RR of hypothyroidism and clinical outcome in HF. Fourth, although we observed little heterogeneity among the studies, the effect of measured (and unmeasured) confounders cannot be avoided in observation studies. Fifth, we had limited information about the use of HF medications such as β-blockers, angiotensin-converting enzyme inhibitors/angiotensin receptor blockers, diuretics, and aldosterone receptor antagonists, which may have contributed to outcomes. Finally, the definition of hypothyroidism varied among studies. We identified a significant difference between studies based on TSH level and a spectrum of thyroid hormone level and found no evidence that hypothyroidism is associated with reduced risk of all-cause death or cardiac death and/or hospitalization.

Even with these limitations, we found no evidence that hypothyroidism is associated with reduced risk of all-cause death or cardiac death and/or hospitalization. Moreover, clinical and biochemical evidence supports a relation between hypothyroidism and outcomes. Our findings should prompt physicians to screen for hypothyroidism, at least TSH levels in HF patients. In addition, the relationship between hypothyroidism and outcomes in HF should be investigated by further studies, including well-designed and carefully controlled cohort studies, to confirm whether the identification of thyroid levels facilitates the early detection of hypothyroidism with a high HF risk.
